# Supplementary material for: Sodium Phenylbutyrate Attenuates Cisplatin-Induced Acute Kidney Injury Through Inhibition of Pyruvate Dehydrogenase Kinase 4
Source: Biomedicines. 2024 Dec 11;12(12):2815. doi: 10.3390/biomedicines12122815 (PMC11672979; doi:10.3390/biomedicines12122815)
Supplement: Supplementary file 1 [file biomedicines-12-02815-s001.zip › biomedicines-3316285-supplementary.pdf]

Supplementary Figure S1

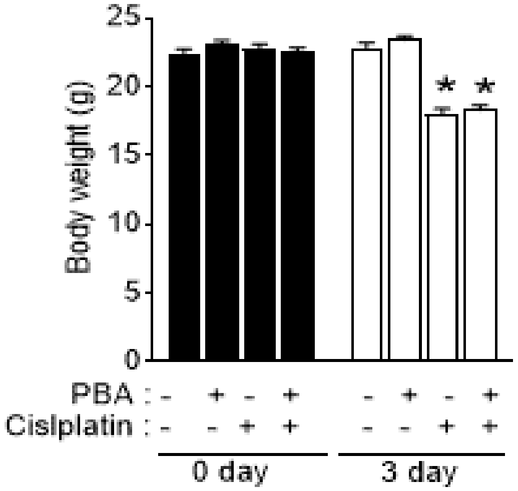

Figure S1. The change in mouse body weight following cisplatin administration.
